# A Systematic Review and Meta-Analysis of the Direct Comparison of Second-Generation Cryoballoon Ablation and Contact Force-Sensing Radiofrequency Ablation in Patients with Paroxysmal Atrial Fibrillation

**DOI:** 10.3390/jpm12020298

**Published:** 2022-02-17

**Authors:** Yoga Waranugraha, Ardian Rizal, Yoga Yuniadi

**Affiliations:** 1 Department of Cardiology and Vascular Medicine, Faculty of Medicine, Universitas Brawijaya, Universitas Brawijaya Hospital, Malang 65142, Indonesia; mr.waranugraha@ub.ac.id; 2 Department of Cardiology and Vascular Medicine, Faculty of Medicine, Universitas Brawijaya, Dr. Saiful Anwar General Hospital, Malang 65111, Indonesia; drardianrizal@ub.ac.id; 3 Department of Cardiology and Vascular Medicine, Faculty of Medicine, Universitas Indonesia, National Cardiovascular Center Harapan Kita, Jakarta Barat 11420, Indonesia

**Keywords:** paroxysmal atrial fibrillation, catheter ablation, second-generation cryoballoon ablation, contact force-sensing radiofrequency ablation

## Abstract

The superiority of second-generation cryoballoon (2G-CB) ablation versus contact force-sensing radiofrequency (CF-RF) ablation in patients with paroxysmal atrial fibrillation (AF) was assessed in this systematic review and meta-analysis. Freedom from atrial tachyarrhythmias (ATAs) (OR = 0.89; 95% confidence interval [CI] = 0.68 to 1.17; *p* = 0.41), freedom from AF (OR = 0.93; 95% CI = 0.65 to 1.35; *p* = 0.72), and acute pulmonary vein isolation (PVI) (OR = 1.17; 95% CI = 0.54 to 2.53; *p* = 0.70) between 2G-CB ablation and CF-RF ablation were not different. The procedure time for the 2G-CB ablation was shorter (MD = −18.78 min; 95% CI = −27.72 to −9.85 min; *p* < 0.01), while the fluoroscopy time was similar (MD = 2.66 min; 95% CI = −0.52 to 5.83 min; *p* = 0.10). In the 2G-CB ablation group, phrenic nerve paralysis was more common (OR = 5.74; 95% CI = 1.80 to 18.31; *p* = < 0.01). Regarding freedom from ATAs, freedom from AF, and acute PVI, these findings imply that 2G-CB ablation is not superior to CF-RF ablation in paroxysmal AF. Although faster than CF-RF ablation, 2G-CB ablation has a greater risk of phrenic nerve paralysis.

## 1. Introduction

In daily clinical practice, the most common arrhythmia encountered by the physician is atrial fibrillation (AF) [1,2]. In 2017, the global prevalence of AF was estimated to be 37.6 million cases, with an increase of more than 60% expected by 2050 [3]. AF is strongly associated with significant mortality, morbidity, and decreased quality of life [4,5,6,7]. Ectopic beats originating from the pulmonary veins (PVs) are responsible for the initiation of paroxysmal AF [8,9]. Based on the latest guidelines from the European Society of Cardiology (ESC), pulmonary vein isolation (PVI) using catheter ablation is recommended for rhythm control strategy [10]. In patients with paroxysmal AF, this has the highest efficacy as a stand-alone procedure [11]. The complete PVI can be achieved by the radiofrequency or cryoballoon ablation procedures. However, several randomized control trials (RCTs) demonstrated conflicting data [12,13,14,15]. A meta-analysis of RCTs revealed equal efficacy between them [16].

Until now, either “freezing” or “burning” approaches are still being debated, and innovations are constantly being made to improve the efficiency and effectiveness of the PVI procedure. The second-generation cryoballoon (2G-CB) catheter was introduced in 2012 to gain more uniform freezing over the whole distal hemisphere of the balloon [17,18]. Compared to the first-generation cryoballoon (1G-CB) catheter, ablation using a 2G-CB catheter demonstrated a similar procedure-related complications rate, reduced fluoroscopy time, shorter procedure time, and higher procedural success rate [19,20]. On the other hand, the contact force-sensing radiofrequency (CF-RF) catheter was released in 2014. It is equipped with the specific ability to measure real-time catheter-tissue contact force to guide ablation more precisely [21,22]. Compared with non-contact force-sensing radiofrequency (non-CF-RF) ablation, CF-RF ablation revealed lower acute PV reconnection [23] and one-year AF recurrence [24]. We needed to know whether 2G-CB ablation was superior to CF-RF ablation for PVI in patients with paroxysmal AF. Therefore, we conducted a systematic review and meta-analysis.

## 2. Materials and Methods

### 2.1. Literature Search

When conducting this systematic review and meta-analysis, the Preferred Reporting Items for Systematic Reviews and Meta-Analyses (PRISMA) statement was followed [25]. Relevant articles comparing 2G-CB ablation with RF-CF ablation for paroxysmal AF recorded in scientific electronic databases such as ClinicalTrials.gov, Cochrane, ProQuest, PubMed, and ScienceDirect were collected and identified according to the eligibility criteria until 31 January 2021. The following keywords were used to find relevant articles: “ablation” or “catheter ablation,” AND “pulmonary vein isolation” or “PVI,” AND “second-generation cryoballoon” or “2nd generation cryoballoon,” AND “contact force radiofrequency,” or “contact force-sensing radiofrequency,” AND “paroxysmal atrial fibrillation” or “paroxysmal AF.” We also gathered and identified potentially relevant papers from the reference lists of the examined articles. Table S1 summarizes the detailed search strategy. The titles, abstracts, and keywords of the identified records were reviewed. Following that, the full texts of all eligible records were examined.

### 2.2. Eligibility Criteria

The inclusion criteria included: (1) RCTs or cohort studies comparing 2G-CB ablation and CF-RF ablation for PVI in paroxysmal AF patients; (2) articles written in English; (3) catheter ablation aimed for rhythm control strategy; (4) sample size of at least 20 patients in each study arm; (5) follow-up duration more than three months; (6) clear information about the arrhythmia detection method; and (7) articles providing detailed relevant data on the outcomes of each study arm. Articles were excluded if they: (1) were duplicates; (2) were sub-studies of the involved studies; (3) included non-paroxysmal AF patients; (4) had incomparable treatment and control groups; (5) did not report the outcomes of interest.

### 2.3. Exposure and Outcomes

Patients were separated into two groups based on the ablation strategy: the “2G-CB group” and the “CF-RF group.” Freedom from atrial tachyarrhythmias (ATAs) after a single catheter ablation procedure was the primary outcome of this study. The secondary outcome involved: (1) freedom from AF after a single catheter ablation procedure; (2) acute PVI after a single catheter ablation procedure; (3) all procedural complications; (4) pericardial effusion/cardiac tamponade; (5) phrenic nerve palsy; (6) vascular complications; (7) procedure time; and (8) fluoroscopy time.

### 2.4. Quality Assessment and Data Extraction

Our study included RCTs and cohort studies comparing 2G-CB ablation and CF-RF ablation. The modified Jadad scale, which comprises eight criteria, was used to assess the quality of RCTs [26]. The total modified Jadad scale ranges from 0 to 8. RCTs with a modified Jadad score of 4 to 8 were considered high-quality [27,28]. For cohort studies, the Methodological Index for Non-randomized Studies (MINORS) was used to assess quality. MINORS has 12 variables [29]. Comparative cohort studies with MINORS scores of 19 to 24 were considered high-quality [30]. All essential information about: (1) the first author name; (2) publication date; (3) study design; (4) 3D mapping system; (5) cryoballoon ablation (CBA) strategy; (6) radiofrequency ablation (RFA) strategy; (7) blanking period; (8) follow-up period; (9) antiarrhythmic drugs (AADs) treatment during follow-up period; (10) arrhythmia detection method; (11) treatment arms; (12) number of patients; (13) age; (14) sex; (15) comorbid diseases such as hypertension, coronary artery disease (CAD), heart failure, sleep apnea, diabetes mellitus (DM), stroke, or transient ischemic attack (TIA); and (16) echocardiographic variables such as left ventricular ejection fraction (LVEF), left atrial volume index (LAVI), and left atrial diameter (LAD), were extracted from each article. The continuous and categorical data were presented as mean ± standard deviation (SD) and number (percentage), respectively. We calculated the mean ± SD from the median and interquartile range (IQR) [31,32].

### 2.5. Statistical Analysis

We followed standard guidelines to conduct the statistical analysis [33]. Heterogeneity among the involved studies was analyzed using Cochran’s Q test and inconsistency index (I^2^). The *p*-value of Cochrane’s Q test < 0.1 or I^2^ > 50% was considered as the presence of heterogeneity [34,35,36]. The pooled effects were determined using a random-effects model because of various study types (RCTs and cohort studies) and the wide range of potential treatment effect sizes across studies [37]. The pooled effects were presented as odds ratio (0R) or mean difference (MD) for dichotomous or continuous outcomes, respectively. We also estimated their 95% confidence interval (CI). Statistically significance was considered to be a *p*-value < 0.05. To find the publication bias, we utilized a mix of Egger’s and Begg’s tests. Egger’s and Begg’s tests revealed publication bias with a *p*-value of 0.05 [38,39,40,41]. The statistical analysis was performed by two investigators using a combination of Review Manager (RevMan) version 5.3 (Cochrane, Copenhagen, Denmark) and Comprehensive Meta-Analysis (CMA) version 3.0 (Biostat, Englewood, NJ, USA).

## 3. Results

### 3.1. Study Selection Process

**Figure 1:**
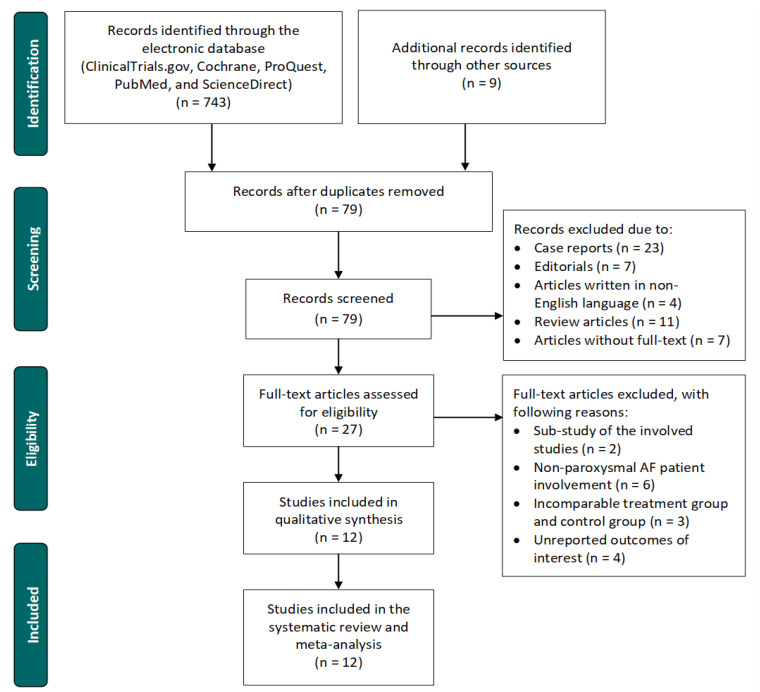
Flowchart of the study selection process. AF = atrial fibrillation.

Of the initial 752 collected articles, 12 studies were eligible to be included in this systematic review and meta-analysis [42,43,44,45,46,47,48,49,50,51,52,53]. A flowchart describing the study selection process is presented in Figure 1. The study quality assessment is shown in Tables S2 and S3.

### 3.2. Baseline Characteristics

Our current systematic review and meta-analysis included one multicenter RCT [42], two single-center RCTs [43,51], six single-center cohort studies [44,45,46,47,50,53], and three multicenter cohort studies [48,49,52]. Electro-anatomical mapping was conducted using CARTO 3 in nine studies [42,43,44,45,46,47,48,51,52]. In three studies, electro-anatomical mapping was performed using CARTO 3 or EnSite [49,50,53]. Only a study from Squara et al. [49] used the 23 or 28 mm 2G-CB catheters. However, in other studies, the 28-mm 2G-CB catheter was used to conduct cryoballoon ablation [42,43,44,45,46,47,48,50,51,52,53]. Cryoballoon ablation procedures were conducted one to two times for each pulmonary vein, with durations ranging from 180 to 240 s. Radiofrequency ablation procedures were conducted using the CF-RF catheter [42,43,44,45,46,47,48,49,50,51,52,53]. In all studies except the studies from Giannopoulos et al. [42] and Matta et al. [48], the pulmonary veins were isolated using the low-power and/or long-duration radiofrequency ablation approach [43,44,45,46,47,49,50,51,52,53]. All included studies had a three-month blanking period [43,44,45,46,47,48,50,52,53], except the studies from Giannopoulos et al. [42], Squara et al. [49], and Watanabe et al. [51]. The shortest follow-up period was six months [42]. Only three studies allowed AAD administration during the follow-up period [45,48,51]. Arrhythmia detection methods in all studies were conducted using ambulatory cardiac monitoring devices [42,43,44,45,46,47,48,49,50,51,52,53]. The baseline characteristics of the included studies are summarized in Table 1.

A total 1419 of patients, including 734 patients in the 2G-CB group and 685 patients in the CF-RF group, were involved in this study. Around 65.3% of the study population were male. The mean age of the patients was 60.8 ± 1.1 years old. The prevalence of comorbid conditions such as hypertension, CAD, heart failure, sleep apnea, DM, and stroke or TIA were 45.6%, 9.9%, 4.0%, 7.4%, 9.1%, and 6.6%, respectively. The mean LVEF was 62 ± 1.3% and the mean LAD was 40.0 ± 1.1 mm. Data on LAVI were available in the study from Jourda et al. [46]. The mean LAVI was 40.7 ± 2.1 mL/m^2^. Table 2 presents the summary of baseline characteristics of patients from the involved studies.

### 3.3. Heterogeneity and Publication Bias

Heterogeneity was found in procedure time and fluoroscopy time (*p*-value of heterogeneity <0.1 and I^2^ > 50%). For the other outcomes, we did not find any heterogeneity. We also did not find any publication bias, as the *p*-values for the Begg’s and Egger’s tests were ≥0.05 for all outcomes of interest (Table 3 and Table 4). Therefore, sensitivity analysis was not conducted.

### 3.4. Primary Outcome

**Figure 2:**
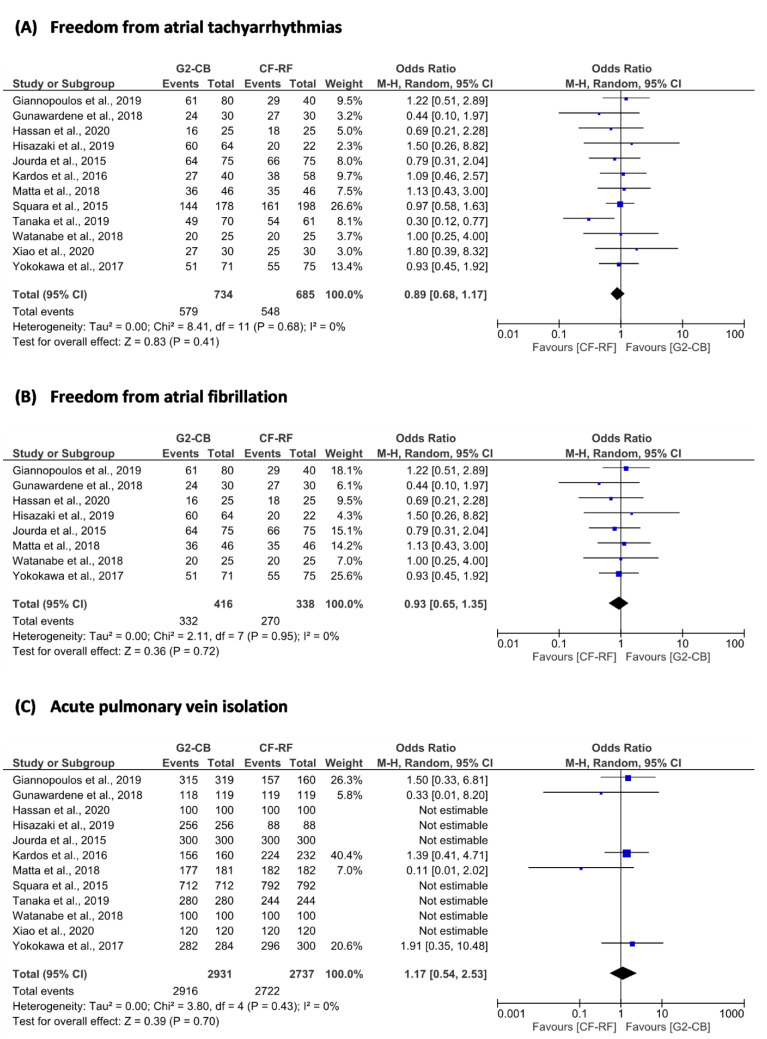
Forest plot of (**A**) freedom from atrial tachyarrhythmias; (**B**) freedom from atrial fibrillation; and (**C**) acute pulmonary vein isolation. 2G-CB = second-generation cryoballoon; CF-RF = contact force-sensing radiofrequency; CI =confidence interval; M–H = Mantel–Haenszel.

The primary outcome of freedom from ATAs was not significantly different between 2G-CB and CF-RF ablation (OR = 0.89; 95% CI = 0.68 to 1.17; p = 0.41) (Figure 2 and Table 3).

### 3.5. Secondary Outcomes

**Figure 3:**
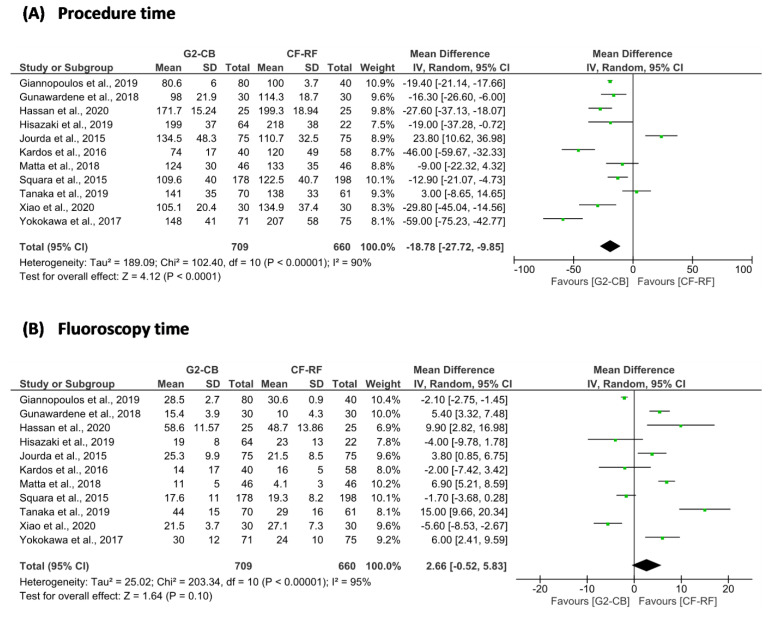
Forest plot of (**A**) procedure time and (**B**) fluoroscopy time; 2G-CB = second-generation cryoballoon; CF-RF = contact force-sensing radiofrequency; CI = confidence interval; IV = inverse variance; SD = standard deviation.

**Figure 4:**
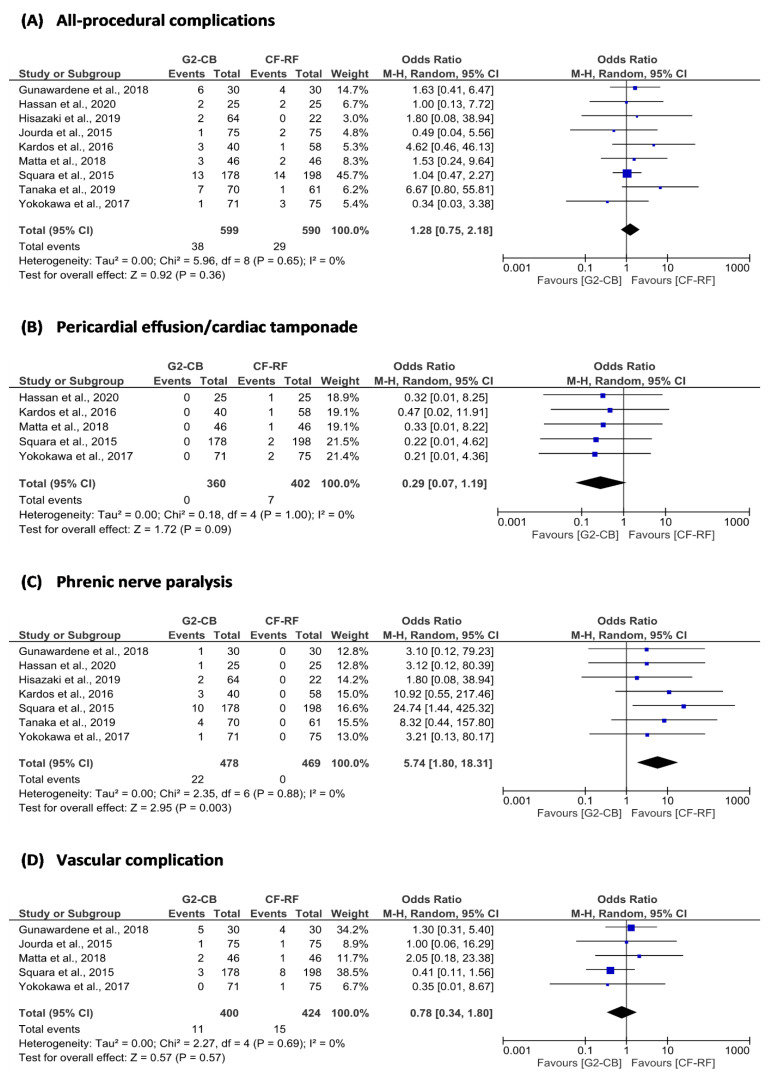
Forest plot of (**A**) all-procedural complications; (**B**) pericardial effusion/cardiac tamponade; (**C**) phrenic nerve paralysis; and (**D**) vascular complications. 2G-CB = second-generation cryoballoon ablation; CF-RF = contact force-sensing radiofrequency ablation; CI = confidence interval; M–H = Mantel–Haenszel.

From the efficacy aspect, we did not find a significant difference in freedom from AF after single ablation procedures between the two groups (OR = 0.93; 95% CI = 0.65 to 1.35; *p* = 0.72) (Figure 2 and Table 3). There was no difference in acute success of PVI between groups (OR = 1.17; 95% CI = 0.54 to 2.53; *p* = 0.70). The procedure time was shorter in the 2G-CB ablation group compared to the CF-RF ablation group (MD = −18.78 min; 95% CI = −27.72 to −9.85 min; *p* < 0.01). However, both groups needed similar fluoroscopy time (MD = 2.66 min; 95% CI = −0.52 to 5.83 min; *p* = 0.10) (Figure 3, Table 3 and Table 4). From a safety aspect, the incidences of all-procedural complications (OR = 1.28; 95% CI = 0.75 to 2.18; *p* = 0.36), pericardial effusion/cardiac tamponade (OR = 0.29; 95% CI = 0.07 to 1.19; *p* = 0.09), and vascular complications (OR = 0.78; 95% CI = 0.34 to 1.80; *p* = 0.57) in both groups were not significantly different. However, 2G-CB ablation was associated with greater incidence of phrenic nerve paralysis (OR = 5.74; 95% CI = 1.80 to 18.31; *p* = < 0.01) (Figure 4 and Table 3).

## 4. Discussion

First, we discovered that 2G-CB ablation for paroxysmal AF was as effective as CF-RF ablation regarding freedom from ATAs, freedom from AF, and acute PVI. Second, even though the fluoroscopy times were comparable, the 2G-CB ablation procedure can be completed faster than the CF-RF ablation procedure. Finally, 2GCB ablation was associated with a greater rate of phrenic nerve paralysis. Furthermore, the 2G-CB group experienced all phrenic nerve paralysis problems.

In today’s paradigm, the electrical isolation of the pulmonary veins from the left atrium is fundamental for most catheter-based ablation strategies in paroxysmal AF. However, there are no specific recommendations from the recent guidelines regarding the choice of CBA or RFA [10,54,55]. CBA and RFA were conducted through femoral access and trans-septal approach. In RFA, operators conduct PVI by point-by-point application of radiofrequency energy under electro-anatomical navigation to generate a contiguous circular lesion surrounding the PV antrum. In CBA, operators conduct PVI by directing the device under fluoroscopic guidance to the PV antrum, advancing it toward the PV, and freezing the surrounding tissue by filling the balloon with a liquid refrigerant [15,56]. RFA results in tissue necrosis by tissue heating, while CBA results in tissue necrosis by the freeze and thaw cycle [57]. PVI using RFA is more complex and time-consuming because it requires complicated catheter manipulations and multiple radiofrequency applications. CBA was developed to simplify the PVI by allowing a single-shot ablation. Compared to the 1G-CB catheter, the 2G-CB catheter has doubled injection ports located more distally in the catheter shaft. This results in a more uniform freezing area on the surface of the balloon [58,59]. On the other hand, the CF-RF catheter is equipped with a contact force sensor on the catheter tip. This can provide important information about the contact force, which is useful for the operator to perform ablation precisely and accurately [60].

At present, the largest RCT comparing CBA and RFA in paroxysmal AF is the FIRE AND ICE trial. This study revealed that CBA was not inferior to RFA regarding efficacy. The overall safety of both procedures was not significantly different. In the FIRE AND ICE trial, the CBA procedures were conducted using 1G-CB or 2G-CB catheters. Moreover, data on CF-RF catheters were not reported in that trial [15]. The FreezeAF study also revealed the non-inferiority of CBA compared to RFA for rhythm control in paroxysmal AF patients [14]. A meta-analysis of RCTs from Murray et al. [16] comparing CBA using 1G-CB or 2G-CB catheters and RFA demonstrated that CBA and RFA had equal efficacy. However, that meta-analysis did not provide information about the use of CF-RF catheters. A meta-analysis from Jiang et al. [61] revealed that 2G-CB ablation effectively decreased the recurrence rate of ATAs compared to RFA in paroxysmal AF patients specifically.

Buist et al. [62] conducted an RCT to compare 2G-CB ablation and CF-RF ablation in AF patients. However, that study included both paroxysmal AF and persistent AF. That study demonstrated that 2G-CB ablation provided better ATA-free survival and lower repeat ablation than CF-RF ablation. The CIRCA-DOSE study revealed that both procedures resulted in similar efficacy for paroxysmal AF during a one-year follow-up duration [63]. However, the study included patients with non-paroxysmal AF in the final analysis. A meta-analysis from Ravi et al., [64] which included RCT and cohort studies comparing CF-RF ablation and 2G-CB ablation, revealed that the efficacy between both groups was similar. Another meta-analysis from Wang et al. [65] that included RCTs showed that AF recurrence rates between both ablation strategies were comparable. However, the meta-analyses from Ravi et al. [64] and Wang et al. [65] involved both paroxysmal AF and persistent AF patients. Compared to the prior meta-analyses, our study specifically compared 2G-CB ablation and CF-RF ablation in patients with paroxysmal AF. Our study also revealed a similar success rate of acute PVI between groups. This result supported the previous study by Wang et al. [65].

Our study demonstrated that 2G-CB ablation in paroxysmal AF could be completed faster than CF-RF ablation. Our result was consistent and supported the previous meta-analyses from Ravi et al. [64] and Wang et al. [65]. 2G-CB ablation can be conducted faster because of its “single-shot” characteristic used throughout the PVI. On the other hand, CF-RF ablation needs a longer procedure time because of its “point-by-point” approach [13]. Previous meta-analyses demonstrated that fluoroscopy time was longer in 2G-CB ablation than in CF-RF ablation [65]. However, in our study, both groups revealed no significantly different fluoroscopy time. We found significant heterogeneity while conducting data analysis of procedure time and fluoroscopy time. That was because of the diverse habits and experience of fluoroscopy utilization among different heart rhythm centers. Increased experience of the operator in performing AF ablation could reduce fluoroscopy time [48]. High power and short-duration (HPSD) radiofrequency ablation procedures are now being conducted to reduce overall procedure time in CF-RF ablation [66]. A study from Baher et al. [67] revealed that compared to the conventional method (35 W power for 10 to 30 s), the HPSD approach (50 W for 5 s) had a shorter procedure time (149 ± 65 min vs. 251 ± 101 min; *p* < 0.001). At present, in paroxysmal AF patients, no study has specifically compared 2G-CB ablation and HPSD CF-RF ablation. Moreover, almost all CF-RF ablation procedures in this meta-analysis were conducted using the conventional method (25 to 35 W power for at least 20 s) [43,44,45,46,47,49,50,51,52,53].

From the safety perspective, our study revealed that 2G-CB ablation and CF-RF ablation did not have significantly different rates of all-procedural complications, pericardial effusion/cardiac tamponade, and vascular complications. Our results supported the findings of prior studies. However, those meta-analyses did not provide data about pericardial effusion/cardiac tamponade and vascular complications [64,65]. Our result revealed that the incidence of pericardial effusion/cardiac tamponade was not significantly different in both groups. However, in a prior meta-analysis from Jiang et al., [61] 2G-CB ablation had a lower rate of pericardial tamponade than RFA (OR = 0.32; 95% CI = 0.13 to 0.78; *p* = 0.01). The possible explanations are: (1) the meta-analysis from Jiang et al. [61] included RFA using the non-CF-RF catheter and CF-RF catheter; (2) our meta-analysis only included CF-RF ablation; (3) the CF-RF catheter provides efficient transfer of heat energy to the ablation target [21]; and (4) controlling radiofrequency power according to contact force appears to prevent or reduce impedance rise, steam pop, and pericardial effusion/tamponade without compromising lesion effectiveness [68]. The risk of phrenic nerve paralysis in our meta-analysis was greater in the 2G-CB group than in the CF-RF group. Our result was similar to and supported the findings of prior meta-analyses [61,64].

We are aware of no other systematic review and meta-analysis of 2G-CB versus RF-CF ablation for individuals with paroxysmal AF. There was no evidence of publication bias in this study. This meta-analysis, on the other hand, has significant limitations that have been highlighted. First, in this systematic review and meta-analysis, RCTs and cohort studies were involved [42,43,44,45,46,47,48,49,50,51,52,53]. Second, data about the specific comorbidities were not always completely available in most studies [42,43,44,45,46,47,49,50,51,52,53]. Third, the definition of freedom from ATAs among the included studies was varied [42,43,44,45,46,47,48,49,50,51,52,53]. Fourth, even though almost all included studies used 12-lead ECG and Holter monitor as the arrhythmia detection methods [42,43,44,45,46,47,48,49,50,51,52], two studies used additional methods such as external loop recorders and auto-triggered event monitors [50,53]. Lastly, there were differences in blanking and follow-up periods duration and the use of AADs during those periods. These limitations could be essential confounders that may have affected the final results.

## 5. Conclusions

In terms of freedom from ATAs, AF, and acute PVI, 2G-CB ablation is not superior to CF-RF ablation in paroxysmal AF. Although the fluoroscopy duration is not significantly different between the two groups, the 2G-CB ablation procedure can be completed faster than the CF-RF ablation procedure. Compared to CF-RF ablation, 2G-CB ablation has a higher rate of phrenic nerve paralysis.

## Figures and Tables

**Table 1:** Baseline characteristics of the involved studies.

Author	Study Design	Mapping System	CBA Strategy	RFA Strategy	Blanking Period	Follow-Up Period	AADs Treatment during Follow-Up Period	Arrhythmia Detection Methods
Giannopoulos et al., 2019 [42]	RCT–MC	CARTO 3	28 mm 2G-CB 240 → 180 s/vein	CF-RF	2 months	6 months	No	12-lead ECG 24 h Holter monitor
Gunawardene et al., 2018 [43]	RCT–SC	CARTO 3	28 mm 2G-CB 1 × 240 s/vein	CF-RF FR 17–30 mL/min Power ≤ 30 W Duration 30–60 s Temperature ≤ 45 °C CF ≥ 10 g	3 months	10.3 ± 2.1 months	No	12-lead ECG 24 h Holter monitor
Hassan et al., 2020 [44]	Cohort–SC	CARTO 3	28 mm 2G-CB 2 × 240 s/vein	CF-RF FR 17–20 mL/min Power 30–35 W Duration 20–40 s FTI > 400 gs	3 months	12 months	No	12-lead ECG 24 h Holter monitor
Hisazaki et al., 2019 [45]	Cohort–SC	CARTO 3	28 mm 2G-CB 2 × 180 s/vein	CF-RF Power ≤ 35 W CF ≥ 10 g	3 months	20 ± 6 months	No/Yes	12-lead ECG 24 h Holter monitor
Jourda et al., 2015 [46]	Cohort–SC	CARTO 3	28 mm 2G-CB 2 × 240 s/vein	CF-RF FR 17–20 mL/min Power ≤ 30 W Temperature ≤ 48 °C	3 months	12 months	No	12-lead ECG 24 h Holter monitor
Kardos et al., 2016 [47]	Cohort–SC	CARTO 3	28 mm 2G-CB ≥1 × 240 s/vein	CF-RF Power ≤ 35 W Duration 20–40 s Temperature ≤ 48 °C	3 months	24 months	No	12-lead ECG 24 h Holter monitor
Matta et al., 2018 [48]	Cohort–MC	CARTO 3	28 mm 2G-CB 180 → 240 s/vein	CF-RF CF 5–15 g	3 months	12 ± 5 months	No/Yes	12-lead ECG 24 to 48 h Holter monitor
Squara et al., 2015 [49]	Cohort–MC	CARTO 3 EnSite	23 or 28 mm 2G-CB 2 × 240 s/vein	CF-RF Power 30–35 W Duration 20–40 FTI > 400 gs	1 months	12 (10–18) months	No	12-lead ECG 24 h Holter monitor
Tanaka et al., 2019 [50]	Cohort–SC	CARTO 3 EnSite	28 mm 2G-CB 2 × 180 s/vein	CF-RF Duration ≥ 20 s CF ≥ 5 g FTI ≥ 150 gs	3 months	2.98 years (median)	No	12-lead ECG Holter monitor External loop recorder
Watanabe et al., 2018 [51]	RCT–SC	CARTO 3	28 mm 2G-CB 2 × 180 s/vein	CF-RF FR 17 mL/min Power ≤ 30 W CF ≥ 10 g	NA	12 months	No/Yes	12-lead ECG 24 to 48 h Holter monitor
Xiao et al., 2020 [52]	Cohort–MC	CARTO 3	28 mm 2G-CB 1 × ≥ 180 s/vein	CF-RF FR 17–25 mL/min Power 25 to 35 W Temperature ≤ 43 °C CF 10–30 g	3 months	12 months	No	12-lead ECG 24 h Holter monitor 7 d Holter monitor
Yokokawa et al., 2017 [53]	Cohort–SC	CARTO 3 EnSite	28 mm 2G-CB 1 × 180 or 240 s/vein	CF-RF FR 30 mL/min Power ≤ 35 W Temperature ≤ 48 °C	3 months	25 ± 5 months	No	Auto-triggered event monitor

AADs = antiarrhythmic drugs; 2G-CB = second-generation cryoballoon ablation; CBA = cryoballoon ablation; CF = contact force; CF-RF = contact force-sensing radiofrequency ablation; ECG = electrocardiogram; FR = flow rate; FTI = force-time integral; MCs = multicenter; NA = not available; RCT = randomized controlled trial; SC = single center.

**Table 2:** Baseline characteristics of the patients from the involved studies.

Author	Group	Patients	Age, Years	Male	Hypertension	CAD	Heart Failure	Sleep Apnea	DM	Stroke or TIA	LVEF, %	LAVI, mL/m^2^	LAD, mm
Giannopoulos, 2019 [42]	2G-CB	80	61.0 ± 2.5	NA	41 (51.3)	6 (7.5)	2 (2.5)	NA	9 (11.3)	NA	59.9 ± 2.3	NA	41.4 ± 4.3
	CF-RF	40	58.3 ± 3.0	NA	18 (45.0)	2 (5.0)	2 (5.0)	NA	6 (15.0)	NA	60.0 ± 2.3	NA	39.9 ± 1.4
Gunawardene, 2018 [43]	2G-CB	30	62.0 ± 9.5	18 (60.0)	16 (53.0)	NA	NA	NA	NA	NA	59.8 ± 4.5	NA	NA
	CF-RF	30	57.4 ± 10.5	24 (80.0)	17 (56.0)	NA	NA	NA	NA	NA	59.2 ± 5.0	NA	NA
Hassan et al., 2020 [44]	2G-CB	25	47.9 ± 11.6	15 (60.0)	6 (24.0)	2 (8.0)	1 (4.0)	NA	7 (28.0)	NA	61.2 ± 5.7	NA	41.0 ± 3.8
	CF-RF	25	45.9 ± 12.4	17 (68.0)	5 (20.0)	1 (4.0)	2(8.0)	NA	5 (20.0)	NA	62.1 ± 7.8	NA	40.9 ± 5.7
Hisazaki et al., 2019 [45]	2G-CB	64	64.0 ± 12.0	40 (63.0)	32 (50.0)	NA	NA	NA	NA	NA	68.0 ± 8.0	NA	35.0 ± 5.0
	CF-RF	22	67.0 ± 12.0	15 (68.0)	10 (45.0)	NA	NA	NA	NA	NA	67.0 ± 8.0	NA	36.0 ± 5.0
Jourda, et al., 2015 [46]	2G-CB	75	59.9 ± 10.6	20 (26.7)	26 (34.7)	NA	5 (6.7)	9 (12.0)	6 (8.0)	3 (4.0)	64.4 ± 7.4	42.8 ± 15.2	NA
	CF-RF	75	62.5 ± 8.9	18 (24.0)	36 (48.0)	NA	2 (2.7)	4 (5.3)	3 (4.0)	8 (10.7)	65.5 ± 5.6	39.5 ± 11.3	NA
Kardos, et al., 2016 [47]	2G-CB	40	59.0 ± 10.0	27 (67.5)	17 (42.5)	5 (12.5)	NA	NA	2 (5.0)	NA	NA	NA	41.3 ± 4.0
	CF-RF	58	61.0 ± 9.0	38 (66.0)	30 (51.0)	7 (12.0)	NA	NA	3 (5.1)	NA	NA	NA	42.1 ± 4.6
Matta, et al., 2018 [48]	2G-CB	46	59.0 ± 9.0	36 (78.0)	21 (46.0)	3 (7.0)	1 (2.0)	2 (4.0)	3 (7.0)	0 (0.0)	61.0 ± 5.0	NA	NA
	CF-RF	46	59.0 ± 9.0	38 (82.0)	21 (46.0)	3 (7.0)	2 (4.0)	3 (7.0)	3 (7.0)	1 (2.0)	61.0 ± 6.0	NA	NA
Squara, et al., 2015 [49]	2G-CB	178	58.4 ± 11.5	128 (71.9)	55 (30.1)	NA	NA	NA	14 (7.9)	NA	56.6 ± 7.7	NA	NA
	CF-RF	198	61.0 ± 9.0	153 (77.3)	74 (37.4)	NA	NA	NA	13 (6.6)	NA	55.8 ± 9.2	NA	NA
Tanaka, et al., 2019 [50]	2G-CB	70	64.1 ± 10.1	52 (74.0)	40 (57.0)	NA	1 (1.0)	NA	7 (10.0)	9 (13.0)	68.0 ± 9.1	NA	37.1 ± 5.7
	CF-RF	61	63.4 ± 10.5	42 (69.0)	38 (62.0)	NA	2 (3.0)	NA	8 (13.0)	4 (7.0)	67.1 ± 6.6	NA	36.9 ± 4.7
Watanabe, et al., 2018 [41]	2G-CB	25	62.0 ± 12.0	17 (68.0)	16 (64.0)	NA	2 (8.0)	NA	3 (12.0)	1 (4.0)	63.0 ± 5.0	NA	39.0 ± 6.0
	CF-RF	25	68.0 ± 9.0	19 (76.0)	14 (56.0)	NA	2 (8.0)	NA	5 (20.0)	2 (8.0)	58.0 ± 8.0	NA	42.0 ± 5.0
Xiao, et al., 2020 [52]	2G-CB	30	64.5 ± 12.1	17 (56.7)	NA	7 (23.3)	NA	NA	NA	NA	63.1 ± 9.6	NA	41.9 ± 5.2
	CF-RF	30	64.1 ± 8.3	19 (63.3)	NA	5 (16.7)	NA	NA	NA	NA	66.4 ± 7.9	NA	40.8 ± 4.9
Yokokawa et al., 2017 [53]	2G-CB	71	63.0 ± 10.0	53 (75.0)	40 (56.0)	10 (14.0)	NA	NA	NA	NA	59.0 ± 6.0	NA	42.5 ± 6.0
	CF-RF	75	62.0 ± 9.0	42 (56.0)	47 (63.0)	5 (6.0)	NA	NA	NA	NA	60.0 ± 5.0	NA	42.5 ± 6.0
Overall		1419	60.8 ± 1.1	65.3	45.6	9.9	4.0	7.4	9.1	6.6	62.0 ± 1.3	40.7 ± 2.1	40.0 ± 1.1

2G-CB = second-generation cryoballoon ablation; CAD = coronary artery disease; CF-RF = contact force-sensing radiofrequency ablation; DM = diabetes mellitus; NA = not available; LA = left atrium; LAD = left atrial diameter; LAVI = left atrial volume index; LVEF = left ventricular ejection fraction; TIA = transient ischemic attack.

**Table 3:** Summary of the primary outcome and secondary outcomes.

Parameters	Number of Studies	2G-CB		CF-RF		Model	OR	95% CI	*p*-Value of Heterogeneity	I^2^ (%)	*p*-Value of Begg’s Test	*p*-Value of Egger’s Test	*p*
		Event, *n* (%)	Total, *n*	Event, *n* (%)	Total, *n*								
Primary outcomes													
Freedom from ATAs	12	579 (78.9)	734	548 (80.0)	685	Random	0.89	0.68 to 1.17	0.68	0	0.73	0.89	0.41
Secondary outcomes													
Freedom from AF	8	332 (79.8)	416	270 (79.9)	338	Random	0.93	0.65 to 1.35	0.95	0	0.71	0.63	0.72
Acute PVI	12	2916 (99.5)	2931	2722 (99.5)	2737	Random	1.17	0.54 to 2.53	0.43	0	0.81	0.08	0.70
All-procedural complications	9	38 (6.3)	599	29 (4.9)	590	Random	1.28	0.75 to 2.18	0.65	0	1.00	0.57	0.36
Pericardial effusion/cardiac tamponade	5	0 (0.0)	360	7 (1.7)	402	Random	0.29	0.07 to 1.19	1.00	0	0.81	0.06	0.09
Phrenic nerve paralysis	7	22 (4.6)	478	0 (0.0)	469	Random	5.74	1.80 to 18.31	0.88	0	0.13	0.07	<0.01
Vascular complications	5	11 (2.8)	400	15 (3.5)	424	Random	0.78	0.34 to 1.80	0.69	0	0.81	0.79	0.57

AF = atrial fibrillation; ATAs = atrial tachyarrhythmia; 2G-CB = second-generation cryoballoon ablation; CI = confidence interval; CF-RF = contact force-sensing radiofrequency ablation; I^2^ = inconsistency index; OR = odds ratio, PVI = pulmonary vein isolation.

**Table 4:** Summary of the procedural time and fluoroscopy time.

Parameters	Number of Studies	2G-CB, *n*	CF-RF, *n*	Model	MD, Minutes	95% CI, Minutes	*p*-Value of Heterogeneity	I^2^ (%)	*p*-Value of Begg’s Test	*p*-Value of Egger’s Test	*p*
Procedure time	11	709	660	Random	−18.78	−27.72 to −9.85	<0.01	90	0.44	0.89	<0.01
Fluoroscopy time	11	709	660	Random	2.66	−0.52 to 5.83	<0.01	95	0.44	0.19	0.10

2G-CB = second-generation cryoballoon ablation; CI = confidence interval; CF-RF = contact force-sensing radiofrequency ablation; I^2^ = inconsistency index; MD = mean difference.

## Data Availability

All data are presented within the article.

## Supplementary Materials

The following are available online at https://www.mdpi.com/article/10.3390/jpm12020298/s1, Table S1: Detailed search strategy, Table S2: Modified Jadad scale, Table S3: Methodological index for non-randomized studies.
