# Supplementary material for: A Systematic Review and Meta-Analysis of the Direct Comparison of Second-Generation Cryoballoon Ablation and Contact Force-Sensing Radiofrequency Ablation in Patients with Paroxysmal Atrial Fibrillation
Source: J Pers Med. 2022 Feb 17;12(2):298. doi: 10.3390/jpm12020298 (PMC8876986; doi:10.3390/jpm12020298)
Supplement: Supplementary file 1 [file jpm-12-00298-s001.zip › jpm-1575304-SI.pdf]

Supplementary Table S1. Detailed search strategy.

Database	Keywords	Record identified
ClinicalTrials.gov	‘pulmonary vein isolation’	199
	‘second-generation cryoballoon’	1
	‘2nd generation cryoballoon’	1
	‘contact force radiofrequency’	12
	‘contact force-sensing radiofrequency’	5
Cochrane	(‘ablation’ OR ‘catheter ablation’) AND (‘pulmonary vein isolation’ OR ‘PVI’) AND (‘second-generation cryoballoon’ OR ‘2nd generation cryoballoon’) AND (‘contact force radiofrequency’ OR ‘contact force-sensing radiofrequency’) AND (‘paroxysmal atrial fibrillation’ OR ‘paroxysmal AF’)	9
ProQuest	(‘ablation’ OR ‘catheter ablation’) AND (‘pulmonary vein isolation’ OR ‘PVI’) AND (‘second-generation cryoballoon’ OR ‘2nd generation cryoballoon’) AND (‘contact force radiofrequency’ OR ‘contact force-sensing radiofrequency’) AND (‘paroxysmal atrial fibrillation’ OR ‘paroxysmal AF’)	290
PubMed	(‘ablation’ OR ‘catheter ablation’) AND (‘pulmonary vein isolation’ OR ‘PVI’) AND (‘second-generation cryoballoon’ OR ‘2nd generation cryoballoon’) AND (‘contact force radiofrequency’ OR ‘contact force-sensing radiofrequency’) AND (‘paroxysmal atrial fibrillation’ OR ‘paroxysmal AF’)	22
ScienceDirect	(‘ablation’ OR ‘catheter ablation’) AND (‘pulmonary vein isolation’ OR ‘PVI’) AND (‘second-generation cryoballoon’ OR ‘2nd generation cryoballoon’) AND (‘contact force radiofrequency’ OR ‘contact force-sensing radiofrequency’) AND (‘paroxysmal atrial fibrillation’ OR ‘paroxysmal AF’)	204
Other source	Potentially relevant papers from the reference lists of the examined articles	9

AF = atrial fibrillation; PVI = pulmonary vein isolation

Supplementary Table S2. Modified Jadad Scale.

**Author**	**Was the study described as randomized?**	**Was the method of randomization appropriate?**	**Was the study described as blinding?**	**Was the method of blinding appropriate?**	**Was there a description of withdrawals and dropouts?**	**Was there a clear description of the inclusion/exclusion criteria?**	**Was the method used to assess adverse effects described?**	**Were the methods of statistical analysis described?**	**Modified Jadad scale**
Giannopoulos, et al, 2019	1	1	0.5	1	0	1	1	1	6.5
Gunawardene, et al, 2018	1	1	0	0	1	1	1	1	6
Watanabe, et al, 2018	1	1	0	0	0	1	1	1	5

Supplementary Table S3. Methodological index for non-randomized studies.

Author	A clearly stated aim	Inclusion of consecutive patients	Prospective collection of data	Endpoints appropriate to the aim the of study	Unbiased assessment of the study endpoint	Follow-up period appropriate to the aim of the study	The loss to follow up <5%	Prospective calculation of study size	An adequate control group	Contemporary group	Baseline equivalence of groups	Adequate statistical analysis	MINORS
Hassan, et al, 2020	2	2	2	2	0	2	2	0	2	2	1	2	19
Hisazaki, et al, 2019	2	2	0	2	0	2	2	0	2	2	2	2	18
Jourda, et al, 2015	2	2	2	2	0	2	0	2	2	2	1	2	19
Kardos, et al, 2016	2	1	2	2	0	2	2	0	2	2	2	2	19
Matta, et al, 2018	2	2	2	2	0	2	0	0	2	2	2	2	18
Squara, et al, 2015	2	2	1	2	0	2	0	0	2	2	2	2	17
Tanaka, et al, 2019	2	2	0	2	0	2	2	2	2	2	2	2	20
Xiao, et al, 2020	2	2	0	2	0	2	0	2	2	2	1	2	17
Yokokawa, et al, 2017	2	2	0	2	0	2	2	0	2	2	1	2	17

MINORS = methodological index for non-randomized studies.
